# Supplementary material for: Shenqi Fuzheng injection reverses M2 macrophage-mediated cisplatin resistance through the PI3K pathway in breast cancer
Source: PLoS One. 2023 Jan 24;18(1):e0279752. doi: 10.1371/journal.pone.0279752 (PMC9873177; doi:10.1371/journal.pone.0279752)
Supplement: S1 Raw images — (PDF) [file pone.0279752.s001.pdf]

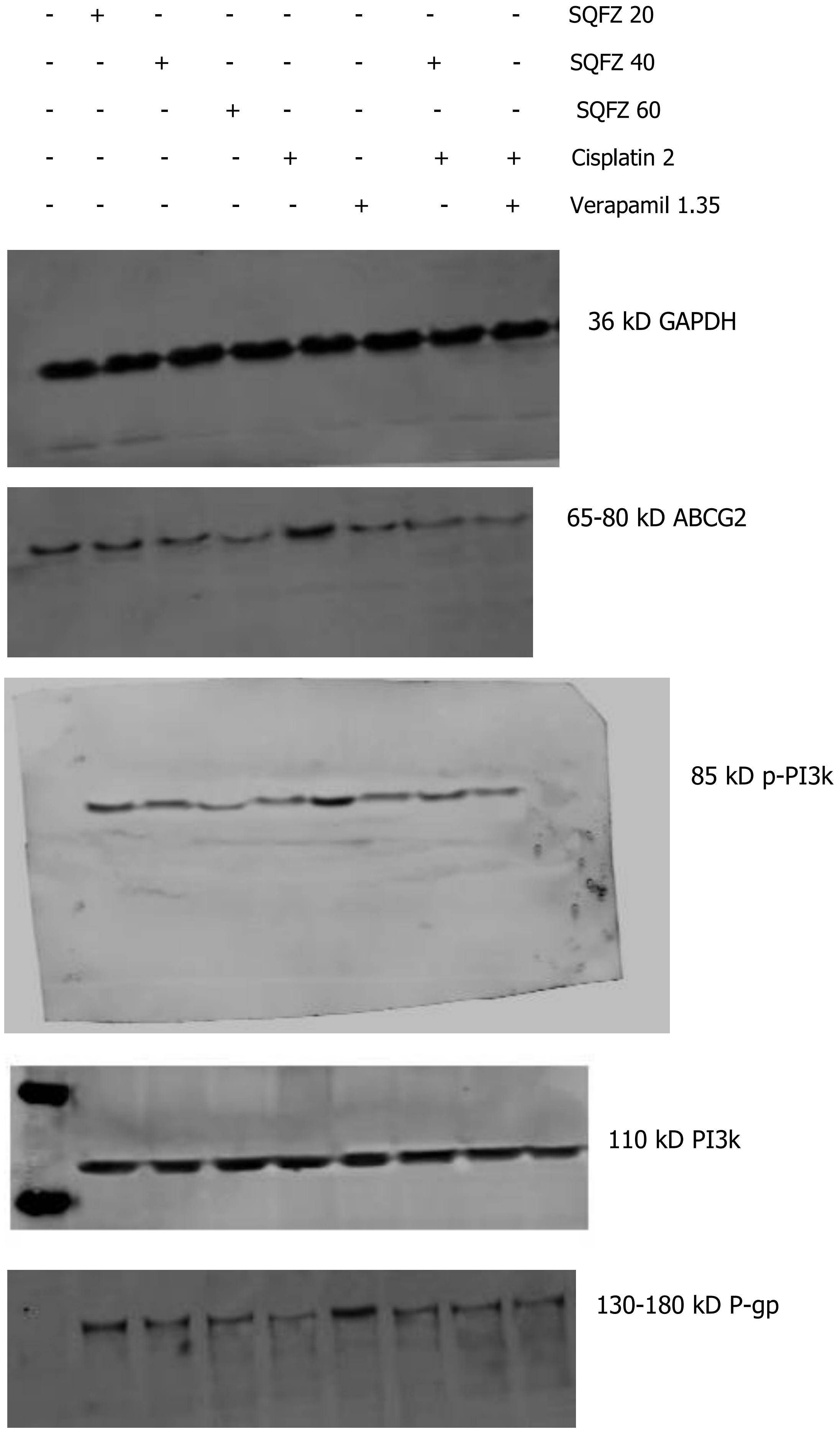
- + - - - - - - SQFZ 20
- - + - - - + - SQFZ 40
- - - + - - - - SQFZ 60
- - - - + - + + Cisplatin 2
- - - - - + - + Verapamil 1.35
36 kD GAPDH
65-80 kD ABCG2
85 kD p-PI3k
110 kD PI3k
130-180 kD P-gp

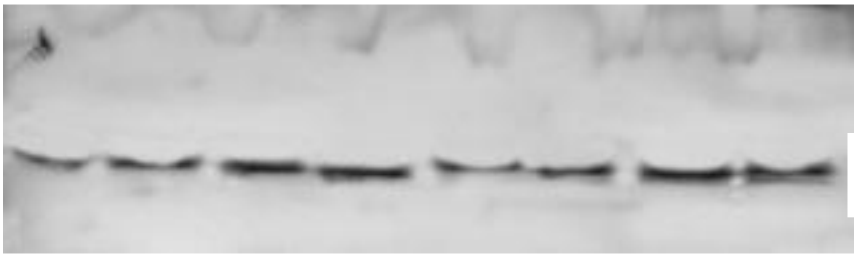

60-85 kD CD86

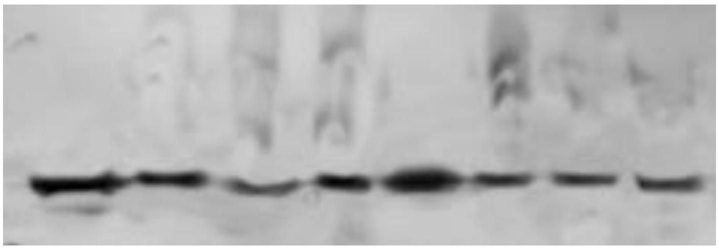

190 kD CD206

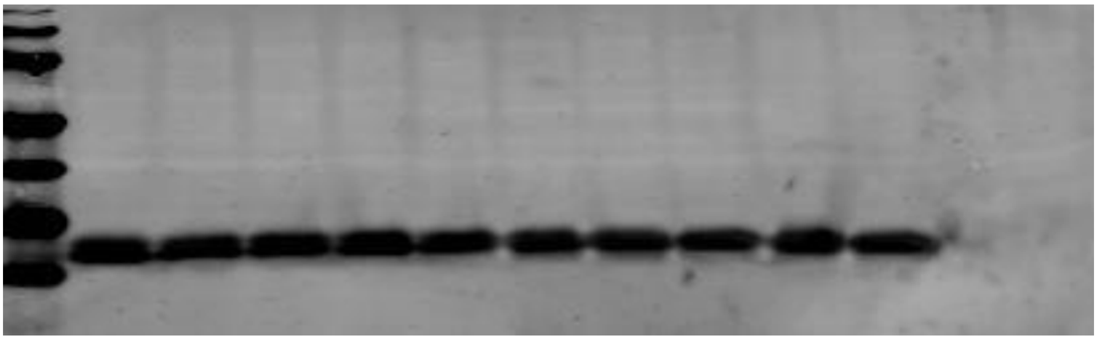

36 kD GAPDH

Control
SQFZ 100 µl/mL
LY294002 20 µmol/L
IGF-1 100 ng/mL
SQFZ+IGF-1

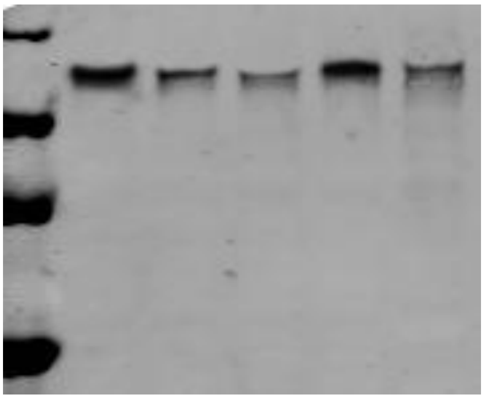

130-180 kD P-gp

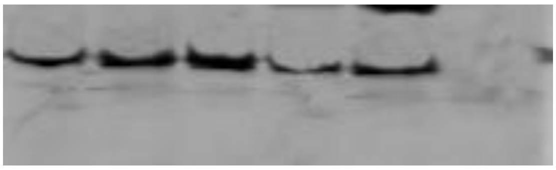

20 kD Bax

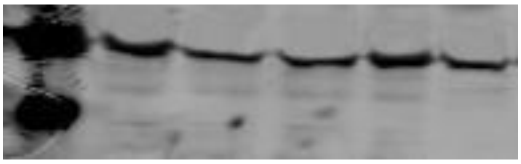

26 kD Bcl-2

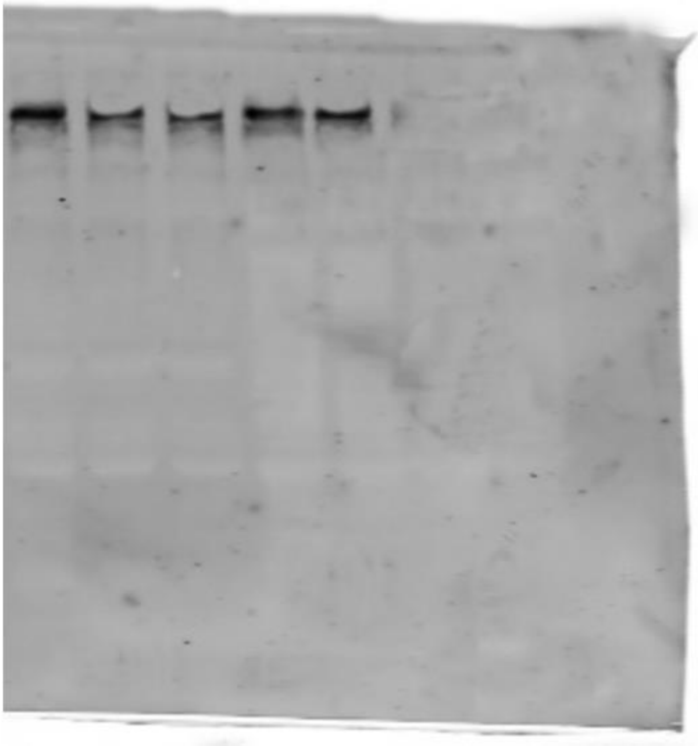

65-80 kD ABCG2

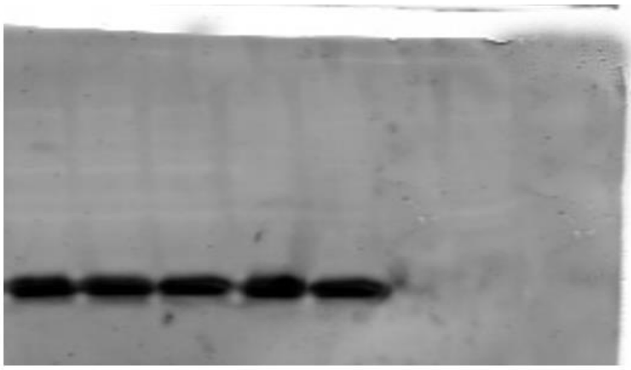

36 kD GAPDH
